# Nested Variant of Urothelial Carcinoma

**DOI:** 10.1155/2014/192720

**Published:** 2014-01-22

**Authors:** Anthony Kodzo-Grey Venyo

**Affiliations:** Department of Urology, North Manchester General Hospital, Delaunays Road, Crumpsall, Manchester, UK

## Abstract

*Background*. Nested variant of urothelial carcinoma was added to the WHO's classification in 2004. *Aims*. To review the literature on nested variant of urothelial carcinoma. *Results*. About 200 cases of the tumour have been reported so far and it has the ensuing morphological features: large numbers of small confluent irregular nests of bland-appearing, closely packed, haphazardly arranged, and poorly defined urothelial cells infiltrating the lamina propria and the muscularis propria. The tumour has a bland histomorphologic appearance, has an aggressive biological behaviour, and has at times been misdiagnosed as a benign lesion which had led to a significant delay in the establishment of the correct diagnosis and contributing to the advanced stage of the disease. Immunohistochemically, the tumour shares some characteristic features with high-risk conventional urothelial carcinomas such as high proliferation index and loss of p27 expression. However, p53, bcl-2, or EGF-r immunoreactivity is not frequently seen. The tumour must be differentiated from a number of proliferative lesions of the urothelium. *Conclusions*. Correct and early diagnosis of this tumour is essential to provide early curative treatment to avoid diagnosis at an advanced stage. A multicentre trial is required to identify treatment options that would improve the outcome of this tumour.

## 1. Introduction

Carcinoma of the urinary bladder is the most common malignancy involving the urinary tract system. Urothelial carcinomas can also occur in the renal pelvis, ureter, or urethra but their occurrence is far less common than in the urinary bladder. The histology of urothelial carcinoma is variable. On the whole about 70% of urothelial carcinomas of the urinary bladder are noninvasive or superficially invasive and these tumours are usually papillary and exhibit different degrees of differentiation; on the other hand, most muscle-invasive urothelial carcinomas are nonpapillary and usually exhibit high-grade cytomorphology. These types of classic urothelial carcinomas can be easily diagnosed histologically and they do not pose a problem to the pathologist.

A number of systems have been utilized to grade and classify urinary bladder tumours. In 1972, the World Health Organization (WHO) adopted a system which distinguished papillomas from grades I, II, and III papillary transitional cell carcinomas. Subsequently in 1998, The World Health Organization in a collaborative effort conjointly with the International Society of Urological Pathologists (ISUP) published a consensus opinion classification system for urothelial (transitional cell) tumours. Studies that were carried out after 1998 were supportive of/validated the clinical significance of the classification scheme, and in view of this in 2004, the classification system was accepted as the standard classification system. According to this classification system, urothelial carcinoma has been classified into (a) low grade and (b) high grade depending upon the degree of nuclear anaplasia and architectural abnormalities with exception of some tumours, for example, tubular or nested/tubular variant. Invasive urothelial carcinoma is of high grade.

A number of variants of urothelial carcinoma were added to the World Health Organization classification in 2004 and some of these include lymphoepithelioma-like cell variant, sarcomatoid variant, plasmacytoid variant, microcystic variant, micropapillary variant, nested variant, and small cell type. These variants of urothelial carcinoma have varied biological behaviours but small cell carcinoma of the urinary bladder is a very aggressive tumour with very poor prognosis. Nested variant of urothelial carcinoma is characterized by an unusual, bland morphology which mimics some benign urinary bladder lesions and it has a clinical behaviour which simulates the clinical behaviour of high-grade conventional urothelial carcinomas. Nested variant of urothelial carcinoma was first reported by Stern [1, 2]. The first reported case of nested variant of urothelial carcinoma was interpreted as a benign lesion, but this lesion subsequently recurred. Pursuant to this Talbert and Young [3] reported 3 cases of nested variant of urothelial carcinoma in 1989 which they described as the carcinomas of the urinary bladder with deceptively benign-appearing foci. Murphy and Deana in 1992 [4] coined for this tumour the terminology of nested variant of transitional cell carcinoma, as it resembles von Brunn's nests. There are reports which indicate that such tumours are diagnosed at an advanced stage and they are associated with inferior prognosis. There is no consensus opinion regarding the optimum management of nested variant of urothelial carcinoma. The ensuing paper contains a review of the literature on nested variant of urothelial carcinoma.

## 2. Methods

Extensive literature search was done using various internet search engines to identify case reports, case series, and review manuscripts as well as conference abstracts on nested variant of urothelial carcinoma using the following terms: nested variant of urothelial carcinoma and nested variant of transitional cell carcinoma. The identified documentations were thoroughly read in order to ascertain the presentation, investigation, diagnostic features, tumour stage, management, and outcome of nested variant of urothelial carcinoma. Details of diagnostic features, tumour details and outcome were not detailed in few of the identified documentations on nested variant of urothelial carcinoma; however, enough information was gathered to summarize the presentation, diagnosis, management, and outcome of patients in most cases (see Tables 1 and 2).

## 3. Results/Literature Review

### 3.1. Definition

Nested variant of urothelial carcinoma is one of the variants of urothelial carcinoma that was added to the WHO classification in 2004. This variant of urothelial carcinoma exhibits a deceptively bland-appearing invasion by nests of cells [5]. They are rare tumours which are composed of irregular and confluent small nests and abortive tubules which are made up of urothelial cells infiltrating the lamina propria or muscularis propria, usually without any evidence of surface epithelium [3]. Nested variant of urothelial carcinoma was first described in 1989 by Talbert and Young [3] who reported the cases of 3 men aged from 53 to 77, and who had carcinoma of the urinary bladder which was characterized by foci with a deceptively benign histologic appearance. In two cases this feature led to a significant delay in the establishment of the correct diagnosis. The diagnostic difficulty in these cases resulted from the resemblance of foci of infiltrating carcinoma von Brunn's nests, cystitis glandularis, cystitis cystica, and nephrogenic adenoma, alone or in combination. The features that helped in distinguishing these foci from benign processes were an irregular distribution, the presence of large numbers of closely packed epithelial aggregates, focal-to-moderate cytologic atypia, and transitions to unequivocal carcinoma. In the third case, the superficial component of a carcinoma closely resembled an inverted papilloma [3].

### 3.2. Epidemiology

Nested variant of urothelial carcinoma usually occurs in men who are older than 60 years which is similar to the occurrence of classic urothelial carcinoma [6].

### 3.3. Age

Nested variant of urothelial carcinomas has been reported in patients aged between 42 years and 90 years.

### 3.4. Clinical Features

Nested variant of urothelial carcinoma is either rare or underreported with a reported incidence of 0.3% of invasive bladder tumours [6]. Lin and associates [7] stated that the nested variants of urothelial carcinoma exhibit aggressive behaviour despite their bland cytologic features. Wasco and associates [8] stated that the clinical outcome of pure or mixed nested variant with usual urothelial carcinoma is similar. Often nested variant of urothelial carcinoma at first presentation is diagnosed in an advanced stage and the tumour often involves the ureteric orifices [6].

It has been stated that the neoplasm resembles von Brunn's nest [4] and it may be misinterpreted as benign [9].

### 3.5. Treatment

Radical surgical resection is the treatment of choice [6].

### 3.6. Macroscopic Features

Quite often there is no evidence of a clearly defined tumour.

### 3.7. Microscopic Features

The described microscopic features of nested variant of urothelial carcinoma include the following.

- Irregular and confluent small nests and abortive tubules are composed of urothelial cells infiltrating the lamina propria or muscularis propria, usually without surface involvement [2].
- The tumour cells usually exhibit mild atypia (mild pleomorphism, slightly increased nuclear/cytoplasmic ratios, occasional prominent nucleoli, and rare mitotic figures) and resemble cystitis glandularis and cystitis cystica [5].
- Deep tumour-stroma interface is jagged and infiltrative [6].
- Often more atypia and focal anaplasia with increasing depth of invasion are one of the features [6].
- Typical urothelial is often present [10].
- Retraction artefact may be seen [6].
- By definition these tumours cannot be high grade or have overlying surface carcinoma *in situ* [6].

### 3.8. Cytology

It has been stated that subtle features are not diagnostic themselves—these subtle features include medium sized round/polygonal cells with abundant, dense, slightly granular basophilic cytoplasm and well-defined cell borders, irregular cell counters, increased nuclear/cytoplasmic ratio, coarse chromatin, and occasional prominent nucleoli [15].

### 3.9. Positive Immunohistochemical Stains

Nested variants of urothelial carcinoma stain positively for the following:

- CK7, CK20, p63, Ki-67, and CK903 [28],
- variable P53 [6].

### 3.10. Negative Immunohistochemical Stains

Nested variants of urothelial carcinoma stain negatively with

- Bc12, EGFR, and PSA [6].

### 3.11. Differential Diagnosis

Some of the listed differential diagnoses of nested variant of urothelial carcinoma include the following.

- Adenocarcinoma: colonic differentiation and more prominent atypia [6].
- Cystitis cystica/cystitis glandularis: this has no atypia and no invasion [6].
- Inverted papilloma: this has no deep invasion [6].
- Nephrogenic metaplasia/adenoma: this usually has papillary component, prominent tubular, or cystic structures lined by single layer of cuboidal cells, no atypia, and no invasion [6].
- Adenocarcinoma of prostate: this is centred in the prostate gland and immunohistochemically stains positively with PSA and PSAP [6].
- Urothelial carcinoma with small tubules: this is an invasive carcinoma with small gland-like spaces lined by urothelial cells without intracellular mucin or columnar lining; some authors have considered this as part of nested variant of urothelial carcinoma [29].
- von Brunn's nests: these have no invasion, no prominent atypia, and no focal anaplasia as stated by some authors [30].

### 3.12. Characteristic Diagnostic Criteria Used to Confirm Nested Variant of Urothelial Carcinoma

Rouse [5] summarized the diagnostic features that could be used to confirm the diagnosis of nested variant of urothelial carcinoma as follows.

1. Infiltrative pattern: it is worth noting that (a) the infiltrative pattern may sometimes be difficult to assess on biopsies that are small; (b) deep foci of classical jagged invasion quite often exist; (c) if present evidence of muscularis propria involvement is definitional (d) the stroma may be desmoplastic or normal.
2. Predominant pattern—variably sized nests are seen and these are most often small sized and fused.
3. Frequent forms of lumens or spaces—(a) the lumens are quite often empty; however, necrotic debris may be found in them or PASd stainable material; (b) the carcinoma cells forming and lining the spaces do not have secretory/glandular cytoplasmic differentiation; (c) the lining cells of the spaces tend to be transitional or squamous PASd negative; (d) There is no absence of goblet cells; and (e) if extensive then the terminology of microcytic urothelial carcinoma can be used.
4. Cytologically predominantly bland—the cytological features of this tumour include the following (a) focal cytologic atypia is almost invariably present but sometimes this is only present in deeper tissues; (b) the overlying mucosa is often normal or there may be a papillary component; (c) nested variant of urothelial carcinoma often involves the ureteric orifices; and (d) despite the bland cytology these tumours are usually aggressive and invasive tumours [5].

### 3.13. Salient Points from Reported Cases and Case Series

Lin and associates [7] stated the following.

- Nested variant of urothelial carcinoma is characterized by confluent small nests and abortive tubules of mildly atypical neoplastic cells infiltrating the lamina propria and/or muscularis propria of the bladder.
- Despite its deceptively bland histomorphologic appearance, the lesion is reported to have an aggressive behaviour. The collective immunohistochemical expression of suppressor genes, growth factor, and proliferation activity marker had not been previously studied in this disease.
- They had stained formalin-fixed, paraffin-embedded archival tissues from 12 cases of nested variant of urothelial carcinoma with monoclonal antibodies to p21, p27, p53, EGF-R, and bcl-2, as well as the proliferation marker MIB-1. They also evaluated the area of predominant immunoreactivity. They also compared the pattern of immunostaining with the clinical parameters.
- p21 was positive in 10 of 12 cases and located at the deepest portion of the tumour in 5 of 10 positive cases. Immunoreactivity for p27 was seen in 11 of 12 cases and limited to the superficial portion of the tumor in 9 of 11 positive cases. Only 3 and 2 of 12 cases were positive for p53 and bcl-2, respectively. MIB-1 immunoreactivity ranged from 2 to 35% of the neoplastic cells, with most tumors showing a proliferation index of >15%. Follow-up ranged from 3 to 30 months (mean, 17.6 months). All patients except one were alive, although three patients developed metastases. Nested variant of urothelial carcinoma is a deceptively benign-appearing neoplasm with potential of deep invasion and metastases. Immunohistochemically, nested variant of urothelial carcinoma shares some features with high-risk conventional urothelial carcinomas, such as loss of p27 expression and high proliferation index. Nevertheless, p53, bcl-2, or EGF-r immunoreactivity is not frequently seen.

Wang and associates [11] reported a case of urothelial carcinoma which had directly involved a pancreatic allograft with metastasis that occurred in a 49-year-old pancreas and kidney transplant recipient. Her initial clinical presentation and findings of computed tomography scan of the abdomen suggested pancreatitis with features worrisome for rejection. A biopsy of her pancreatic allograft was obtained and histological examination of the specimen revealed that the specimen contained poorly differentiated carcinoma and cystoscopic biopsy disclosed an invasive high-grade urothelial carcinoma arising in the background of extensive urothelial carcinoma *in situ*. She underwent exploratory laparotomy which revealed extensive tumor invading the right ovary and tube, the caecum, and the transplant pancreas with extensive retroperitoneal involvement. Subsequently, she underwent en bloc resection of distal ileum and caecum, resection of transplanted pancreas, partial cystectomy, right salpingo-oophorectomy, and repair of ileocolostomy anastomosis. Pathological examination of the resected specimen disclosed a 4.9 cm mass within the bladder cuff near the allograft that directly invaded the right ovary, fallopian tube, caecum, and pancreas allograft, as well as extensive retroperitoneal involvement. The tumour demonstrated a prominent nested growth pattern reminiscent of the nested variant of urothelial carcinoma (NVUC) with other areas showing features more typical of conventional invasive high-grade urothelial carcinoma. The neoplastic cells were positive for pancytokeratin and OC125 (cytoplasmic) while being negative for chromogranin, synaptophysin, CD56, CK7, CK20, CDX2, TTF1, ER, PR, p53, and BRST2. While not entirely specific, the staining pattern combined with the presence of adjacent urothelial carcinoma *in situ* was supportive of a urothelial origin. In addition, the lesions resected from her abdominal wall were positive for metastatic urothelial carcinoma. Postoperatively, she received four cycles of gemcitabine and carboplatin, which she completed, with no measurable disease noted radiographically following therapy. One year later, she was admitted to hospital for the worsening abdominal pain. She had a computed tomography (CT) scan which revealed multiple intra-abdominal and peritoneal nodules consistent with metastatic disease. She went into a hospice and died shortly after her admission to the hospice.

Wang and associates [11] stated that carcinoma of the urinary bladder developing in organ transplant recipients remains a challenging disease to manage as it has been demonstrated by some authors [33–36] that the clinical course seems worse than that in the general population as reported in [33–36]. Other authors stated that the immunosuppressed status of the transplant recipients renders the therapy and posttreatment surveillance very difficult [37]. With the increase of organ transplantation, urological cancer (including bladder cancer) may pose a critical problem affecting the survival of these patients.

Nested variant of urothelial carcinoma was classified by the World Health Organization in 2004 as an “uncommon aggressive tumor,” with few reported cases and a 70% mortality rate 4 to 40 months after diagnosis despite therapy [38]. Holmäng and Johansson [20] stated that the incidence of nested variant of urothelial carcinoma has been estimated to be 0.8% of all invasive bladder carcinoma [20] and less than 100 cases had been reported [4, 9, 29, 31]. Liedberg and associates [31] stated that nested variant of urothelial carcinoma exhibits aggressive clinical behaviour with rapid spread along the lymphatics in the lamina propria of the urinary bladder and along lymphatic channels into the peritoneum [4, 9, 29, 31].

While the degree of cytologic atypia noted in this case is not typically described in NVUC, this feature can be seen in these lesions and NVUC is associated with areas of conventional high-grade urothelial carcinoma in the majority of instances [8]. In the present case, her pancreas is bladder drained and it is possible that the atypia noted may be related to the effect of exocrine pancreatic secretions. Indeed, clinical behaviour and pattern of spread are compatible with NVUC and cases with nested features have a poor outcome [8].

Wang and associates [11] stated that to their knowledge, their case was the first case of urothelial carcinoma demonstrating NVUC features reported in the transplant receipt; the tumour invaded the transplanted pancreas with extensive retroperitoneal involvement. This is a unique presentation that clinically mimicked pancreatitis and/or rejection. The rapid progression from a clinically nonapparent lesion widely invasive disease may be related to the patient's immunosuppressive status, although as noted above lesions with nested growth patterns often demonstrate an aggressive phenotype.

Some authors [7, 20] stated that the optimal modality of treatment of nested variant of urothelial carcinoma is uncertain because nested variant of urothelial carcinoma is rare and there had not been any randomized studies specifically designed for this subtype of bladder tumour [7, 20]. Sternberg and associates [39] stated that traditionally, the standard therapy for patients with locally advanced or metastatic urothelial carcinoma is chemotherapy using methotrexate, vinblastine, doxorubicin, and cisplatin (MVAC) [39]. von der Maase and associates [40] had shown that the gemcitabine-cisplatin regimen has equivalent overall response rates, with less toxicity (range 41% to 57%), with a complete response in 15% to 22%, and a median survival of 12.5 to 14.3 months [40]. Wang and associates [11] stated that even though a number of authors were of the opinion that this subtype of urothelial carcinoma is resistant to radiotherapy and chemotherapy [7, 20, 31], clinical experience with their case would suggest that multimodality therapy including platinum based chemotherapy is beneficial. Wang and associates [11] suggested that multi-institution studies are needed to establish a better therapeutic protocol for these rare cases. Wang and associates [11] concluded that their case report illustrates atypical presentation of bladder cancer in a pancreas and kidney transplant recipient and that their experience should alert physicians and radiologists to the possibility of malignancy in the differential diagnosis and the need for early biopsy to avoid diagnostic confusion with graft rejection.

Wang and associates [11] stated that postoperatively, the patient was treated with four cycles of carboplatin and gemcitabine. She ultimately succumbed to her disease approximately 1 year after diagnosis. Wang and associates [11] iterated that this case should alert physicians and radiologists to be aware of atypical presentation of urothelial carcinoma in bladder-drained pancreas grafts, the aggressiveness of such lesions, and the need for early biopsy to avoid diagnostic confusion with rejection.

Cox and associates [32] reported 23 cases of large nested variant of urothelial carcinoma from the consult files of one of the authors from 2001 to 2010. They reported that the mean patient age was 63.7 years with an age range from 39 years to 89 years, and 86% were men. Out of the 23 cases, 18 of the patients underwent transurethral resection of bladder tumour, 2 underwent nephroureterectomy, and 3 had undergone radical cystectomy. Cox and associates [32] reported that a surface component was present in 19 of the 23 cases, with 16 low-grade papillary urothelial carcinomas, 2 low-grade papillary urothelial carcinoma with less than 5% high-grade urothelial carcinoma, and 1 high-grade papillary urothelial carcinoma. They reported that, out of the 23 cases, twenty had invaded into the muscularis propria. With regard to 21 cases, the invasive component was found to be composed of medium to large nests which varied from rounded circumscribed borders to stromal-tumour interface with a more irregular ragged appearance. Two of the tumours exhibited a verruciform, pushing border into the muscularis propria with the nests having central cyst formation. With regard to the cytological characteristics, the nuclei lacked significant nuclear atypia, where at most occasional scattered slightly enlarged, hyperchromatic nuclei with small-indistinct nucleoli were noted. Four of the cases had focal necrosis and 3 of the cases had more extensive necrosis. Cox and associates [32] also reported that the median mitotic count was 1.5 per 10 high-power fields. The stroma which surrounded the large nests characteristically had a mild-to-moderate fibrous and/or inflammatory reaction; 4 of the cases did not have any stromal reaction, but 2 cases had a moderate-to-marked stromal response. In 7 of the 23 cases, conventional patterns of urothelial invasion were found, 5 of which consisted of ≤5% of the neoplasm. One of the cases had angiolymphatic invasion. Four cases had subsequent radical cystectomy specimens available for review. Two out of the 4 radical cystectomy specimens did not have any residual carcinoma (1 with neoadjuvant radiotherapy); 1 had large nested urothelial carcinoma in the muscularis propria into the perivesical tissue. Cox and associates [32] reported that clinical follow-up was available for 17 of 23 patients with a mean follow-up of 43 months and a follow-up range of 5 months to 9 years. Cox and associates [32] additionally reported the following.

Three of 17 patients developed metastatic disease (2 in the lung, 1 unknown) with 2 of these dead due to disease; another patient died of disease with un known details. Of the aforementioned 3 patients who died of disease, 2 had no and 1 had focal (<5%) conventional invasive urothelial carcinoma on transurethral resection. Cox and associates [32] stipulated the following.

- These cases, which posed great diagnostic difficulty both for the contributing pathologists and for the consultant, represent the first formal description of a large nested pattern of urothelial carcinoma.
- This pattern is distinguished from an inverted growth pattern of noninvasive urothelial carcinoma and from von Brunn nests by either muscularis propria invasion, irregularly infiltrating nests, or a stromal reaction.
- Despite the bland cytological features of these neoplasms, they have well-documented metastatic potential.

Wasco and associates [8] stated that nested variant of urothelial carcinoma is a rare histological variant of urothelial carcinoma which is characterized by deceptively bland histologic features that resemble von Bruun's nests but usually with a poor outcome. They also stated that in their experience, nested variant of urothelial carcinoma is frequently misclassified or underrecognized in view of the fact that its clinicopathological spectrum is not well defined. Furthermore, its relationship to the usual urothelial carcinoma and response to traditional bladder cancer management are largely unknown. Wasco and associates [8] reported 30 cases of with pure or predominant nested morphology in order to identify its associated histopathological findings, clinical outcome, and immunophenotype. Wasco and associates [8] reported that the ages of the patients ranged from 41 years to 83 years with an average age of 63 years, with a male to female ratio of 2.3 : 1. They also reported that the architectural pattern of the nested component ranged from a predominantly disorderly proliferation of discrete, small, variably sized nests (90%) to focal areas demonstrating confluent nests (40%), cord-like growth (37%), and cystitis cystica-like areas (33%) to tubular growth pattern (13%). The deep tumour-stroma interface was invariably (100%) jagged and infiltrative. Additionally, Wasco and associates [8] stated that despite the overall bland cytology, the tumour nests exhibited focal random cytologic atypia (90%) and focal high-grade cytologic atypia which was centred within the base of the tumour (40%). The tumour stroma ranged from having minimal stromal response to focally desmoplastic and myxoid. They found a component of usual urothelial carcinoma in 63% of cases. Wasco and associates [8] furthermore made the following ensuing reports.

- The nested component demonstrated an immunophenotype which was identical to the usual urothelial carcinoma, with CK7, CK20, p63, and CK903 expression in 93%, 68%, 92%, and 92% of cases, respectively.
- At resection, all of the cases except 1 case were demonstrated to be invasive—9% into lamina propria, 4% into muscularis propria, 65% into perivesical fat, and 17% into adjacent organ(s).
- In comparison with pure high-grade urothelial carcinoma, nested variant of urothelial carcinoma was associated with muscleinvasion at transurethral resection (31% versus 70%; *P* < 0.0001), extravesical disease at cystectomy (33% versus 83%, *P* < 0.0001), and metastatic disease (19% versus 67%, *P* < 0.0001).
- Follow-up was available for 29 patients (97%) with a median of 12 months (range, 1 month to 31 months) of follow-up; 3 (10%) died of disease, 16 (55%) were alive with persistent or recurrent disease, and 10 (34%) were alive without disease.
- Response to neoadjuvant chemotherapy was observed in 2 (13%) of 15 patients.
- Nested variant of urothelial carcinoma which was seen either in pure form or with a component of usual urothelial carcinoma had similarly unfavourable outcome (*P* = 0.78).

Wasco and associates [8] concluded that increased awareness and familiarity with the clinicopathologic spectrum of nested variant of urothelial carcinoma is critical for confident recognition and adequate management of this very aggressive variant of urothelial carcinoma.

Dundar and associates [16] reported two cases of nested variant of urothelial carcinoma. In the first case the tumour extended through the bladder wall into the perivesical soft tissue, prostatic urethra, and left seminal vesicle and metastasized to the obturator lymph nodes. In the second case invasion of muscular layer was observed and three recurrences were developed during a follow-up period of 23 months. Dundar and associates [16] stated that both tumours demonstrated high p53 and Ki-67 indices supporting the aggressive nature of such tumours. Details of the two reported cases are as follows.

**Figure 1:**
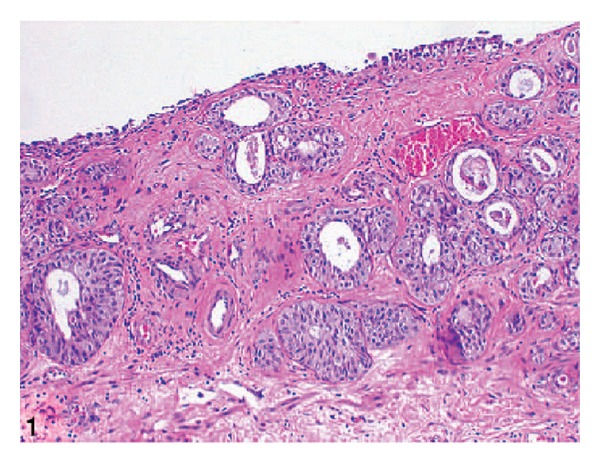
Low-power view showing closely packed, irregularly spaced, glandular, and cystic urothelial nests somewhat resembling von Brunn nests. Note that the overlying urothelium appears uninvolved (hematoxylin-eosin, original magnification ×100). Nested variant of urothelial carcinoma taken from Dhall et al. [2]. The figures have been reproduced with the permission of the editor in chief of Archives of Pathology and Laboratory Medicine on behalf of the editorial board of the journal.

**Figure 2:**
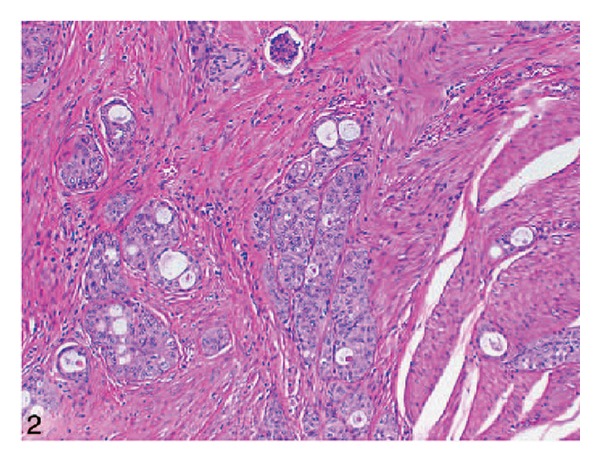
Low-power view showing bland tumor cells in the nested pattern infiltrating the muscularis propria (hematoxylin-eosin, original magnification ×100). Nested variant of urothelial carcinoma taken from Dhall et al. [2]. The figures have been reproduced with the permission of the editor in chief of Archives of Pathology and Laboratory Medicine on behalf of the editorial board of the journal.

*Case 1.* A 70-year-old man presented with symptoms of urinary urgency, increased frequency, and nocturia of one-year duration. He had an ultrasound scan and this revealed a 5 cm diameter polypoid mass in the left posterolateral wall of the bladder. The mass was located at close proximity to the left ureteric orifice and this was associated with left sided hydronephrosis. He underwent transurethral resection of the bladder lesion as well as lesion within the prostatic urethra. Histological examination of the specimen revealed small, closely packed nests of epithelial cells infiltrating the lamina propria and muscularis propria of the bladder wall (Figure 1). The tumour cells were observed to be uniform with only focal moderate atypia. Tumoral invasion was also seen in the prostatic urethra. He had computed tomography scans of abdomen and thorax as well as scintigraphic examination which demonstrated that there was no metastatic disease. He then underwent radical cystoprostatectomy. Microscopic examination revealed that the tumour had extended through the bladder wall into the perivesical soft tissue, prostatic urethra, and left seminal vesicle. The neoplastic cells characteristically exhibited pale, eosinophilic, or clear cytoplasm and rounded nuclei with inconspicuous nucleoli. The cells were reported to have shown generally mild atypical features but occasionally large atypical cells were observed in the deeply infiltrated areas (Figure 2). Extensive perineural invasion was observed. Additionally, two out of 14 and 2 out of 11 right and left obturator lymph nodes were positive for the tumour, respectively. The iliac lymph nodes were negative for the tumour. He was scheduled to receive systemic chemotherapy which he had not yet received at the time of publication of the paper and therefore there was no follow-up outcome data available.

*Case 2.* A 56-year-old man had been followed up (for 10 years—since 1996) for a WHO grade 2 papillary urothelial carcinoma which was localized towards the left lateral wall of the urinary bladder. At a routine follow-up cystoscopic examination, two small tumour foci in the dome of the urinary bladder were found which were resected transurethrally. The microscopic features of the tumour were markedly different from those of his previous tumour by the infiltration of neoplastic cells that were arranged in a diffuse pattern of variably sized nests. There was evidence of invasion of lamina propria and muscular layer. There was no evidence of papillary configuration in the tumour. The tumour cells characteristically exhibited pale to eosinophilic cytoplasm with bland nuclear features. The mitotic activity in the tumour was low, and there was no evidence of perineural invasion. The histological findings were reported to be consistent with those of nested variant of urothelial carcinoma. The option of radical cystoprostatectomy was offered to the patient but he refused to undergo cystoprostatectomy. However, during a follow-up period of 23 months, he developed three recurrences of nested variant of urothelial carcinoma.

**Figure 3:**
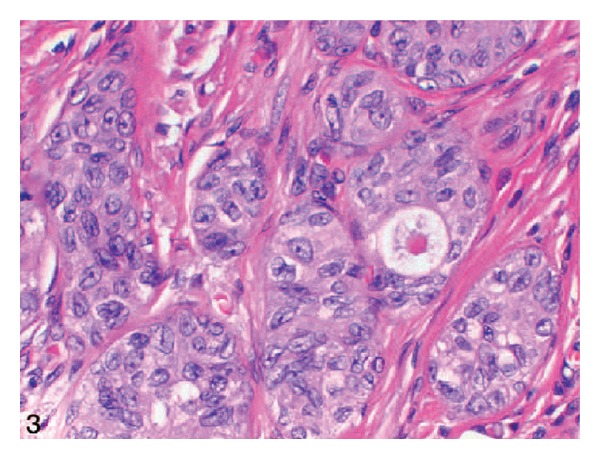
On high-power view, the tumour cells show no significant cytologic atypia (hematoxylin-eosin, original magnification ×400). Nested variant of urothelial carcinoma taken from Dhall et al. [2]. The figures have been reproduced with the permission of the editor in chief of Archives of Pathology and Laboratory Medicine on behalf of the editorial board of the journal.

Immunohistochemical studies of the tumour were undertaken and these showed that the percentages of cells that were positive for Ki-67, p53, and p27 were 20%, 40%, and 40% in Case  1 and 15%, 40%, and 50% in Case  2, respectively (Figure 3). In both Cases  1 and  2, the tumour cells were positive for high-molecular-weight cytokeratin (34*β*E12) but negative for PSA and low-molecular weight cytokeratin (AE1).

Terada [13] in a report of two cases stated that the nested variant of urothelial carcinoma is characterized by the presence of benign appearing urothelial carcinoma cells in the lamina propria, sparing the surface urothelial involvement, and that the tumour exhibited aggressive clinical course despite having a benign-looking histological appearance. Terada [13] reported two cases one in an 80-year-old woman and the second in a 78-year-old man. Terada [13] reported that in both cases atypical cells forming nests and tubules were observed in the lamina propria without the involvement of surface urothelium. Additionally, Terada [13] reported that one case resembled nephrogenic metaplasia and another resembled proliferated von Brunn's nest or inverted papilloma. Terada [13] reported the results of immunohistochemical studies on both tumours as follows.

- Both cases were positive with P53 and high Ki7 labeling, which suggested that both cases were malignant.
- One case was characterized by positive cytokeratins, EMA, p53, Ki-67 (labeling = 15%), CD10, CEA, and MUC1.
- The patients were free of tumour 6 months and at 15 months pursuant to transurethral resection of their bladder tumours.

Cardillo and associates [15] stated that in view of the fact that nested variant of urothelial carcinoma is a recently described rare variant of urothelial carcinoma there had not been any prior report of cytologic findings in urine specimens from patients with nested variant of urothelial carcinoma. Cardillo and associates [15] reviewed urine specimens from patients with histologically confirmed nested variant of urothelial carcinoma. They evaluated urine specimens that were obtained concurrently with or up to 1 month prior to the patient's surgical procedure. They analysed all the specimens for the presence of cells morphologically similar to the nested variant of urothelial carcinoma cells that were observed in the tissue sections. The cells were observed for the ensuing parameters: the number of neoplastic cells; the cellular arrangement, the cell size and shape, cell borders, as well as cytoplasmic, nuclear, and nucleolar characteristics. They included thirteen urine specimens from 7 patients in the study. Cardillo and associates [15] reported that they were able to identify cells that were similar morphologically to cells present in the nests of nested variant of urothelial carcinoma in all cytologic specimens. They iterated that the neoplastic cells for most part were medium sized, round, or polygonal, with abundant, dense, slightly granular basophilic cytoplasm, and well-defined cell borders. The nuclear/cytoplasmic ratio was increased, the nuclear membranes exhibited irregular contours, and the nuclei encompassed coarse chromatin with occasional prominent nucleoli. Cardillo and associates [15] made the following concluding iterations.

- The cytologic features of nested variant of urothelial carcinoma are subtle but distinct.
- A primary diagnosis of nested variant of urothelial carcinoma is not recommended in view of the subtleness of the findings.
- However, the presence of cells with the aforedescribed features should warrant a cystoscopic examination with histological confirmation in a patient with a previous history of nested variant of urothelial carcinoma.

De Berardinis and associates in 2012 stated that nested variant of urothelial carcinoma is a rare histological entity, with about 80 reported cases [14]. De Beradinis and associates [14] reported the case of 70-year-old man with haematuria who underwent ultrasound scan and cystoscopy which revealed the presence of carcinoma of the urinary bladder. He underwent transurethral resection of the tumour and histology of the resected tumour revealed a high-grade cancer with lamina propria involvement (G3pT1). He had adjuvant intravesical instillation of Bacillus Calmette-Guérin and at follow-up cystoscopy five months later a recurrent tumour was found in the bladder and this tumour was resected. Histological examination revealed a high-grade (G3) nested variant of transitional cell carcinoma with a deep lamina propria involvement (pT1b). Immunohistochemical examination of the tumour revealed high expression of tumour suppressor gene p53 and immunoreactivity for Ki-67. He then underwent radical cystectomy and histological examination of the specimen revealed a mixed urothelial nested variant tumour which was staged pT2a and grade G3 (poorly differentiated) with lymphatic involvement. Twelve months after the first diagnosis of the bladder cancer, he underwent a cycle of intravenous gemcitabine along with cisplatin. The case was reported just after the operation, therefore, there was no follow-up information on the patient's follow-up outcome. However, De Berardinis and associates [14] were of the opinion that the aggressive behaviour of this neoplasm would suggest that the correct indication for its treatment should be early radical cystectomy with extended lymph adenectomy in order to avoid the progression of the tumour into the urinary bladder wall or metastatic spread. They also stated that it is important to bear in mind that Ki-67 expression that is low in benign lesions and high in nested variant of bladder cancer can be considered a good method to distinguish between the two entities.

Xiao and associates [28] reported 2 cases of nested variant of urothelial carcinoma; the patients in both cases were elderly men, with a predominant involvement of the trigone. Microscopic examination revealed that the tumour cells were arranged in ill-defined nests and had low-grade nuclear features. Both cases had a diffusely infiltrating growth pattern with widespread local disease at cystectomy. Strong immunohistochemical staining for p63 in the neoplastic cells supported the urothelial cell nature of this neoplasm. High p53 and Ki-67 indices of the tumour correlated with the aggressiveness of this subtype of urothelial carcinoma. Details of the two cases were as follows.

*Case 1.* A 69-year-old man presented with 2 episodes of visible haematuria. He had computed tomography scan which revealed diffuse thickening of the bladder wall and moderate hydronephrosis. There was no evidence of lymphadenopathy or metastatic disease. He did not have urine cytology examination. He underwent cystoscopy and bladder biopsy from the trigone and histological examination of the specimen revealed muscle-invasive urothelial carcinoma involving the trigone. Immunohistochemical staining of the tumour revealed immunoreactivity for high-molecular weight cytokeratin 903 and cytokeratins 7 and 20 and negativity for prostatic specific antigen and prostatic acid phosphatase. He underwent radical cystoprostatectomy. Macroscopic examination revealed that the mucosa of the urinary bladder was oedematous, with focal haemorrhage and necrosis around the trigone. The urinary bladder wall was diffusely thickened and infiltrated by tumour; the trigone of the urinary bladder was most markedly involved, with obstruction of the ureteric orifices (see Figure 5). Microscopic examination revealed that with the exception of the surface mucosa, the bladder wall was extensively infiltrated by neoplastic cells, which were arranged in a diffuse pattern of relatively ill-defined and variably sized nests (see Figures 6 and 7). There was also evidence of focal urothelial carcinoma *in situ* and multiple foci of urothelial dysplasia. The cytologic characteristics of the underlying main neoplasm were markedly distinct from those of the surface urothelial mucosal lesions. The neoplastic cells exhibited clear/pale or amophilic/eosinophilic cytoplasm with poorly defined borders and rounded nuclei with inconspicuous nucleoli. The chromatin was finely granular and distributed in an even fashion. Rarely, the tumour cells exhibited clear and signet ring characteristics. On the whole, there was no evidence of significant nuclear pleomorphism, and mitosis was rare (see Figure 7). Rarely, large atypical cells were observed in the deeply infiltrated area (muscularis propria and fat). Within the infiltrating tumour, a prominent desmoplastic reaction was conspicuous. The main tumour had extended through the vesical wall into the perivesical soft tissue and this had also involved the prostatic urethra and paraurethral tissues. There was evidence of metastatic carcinoma in the perivesical lymph nodes. Additionally, bilateral obturator and iliac lymph nodes did not contain any metastasis. Nevertheless, metastasis was found in the adjacent obturator adipose tissue. A diagnosis of high-grade nested variant of urothelial carcinoma was made. He subsequently received adjuvant chemotherapy with taxol and carboplatin. He developed bone and soft tissue metastases four months pursuant to resection of his tumour.

*Case 2.* A 70-year-old man was investigated 3 years earlier when he presented with visible haematuria urinary frequency and occasional straining to void. His repeated urine cytology and intravenous urography were normal and his cystoscopic examination revealed a focal area of localized inflammation on the floor of the urinary bladder lateral to the right ureteric orifice. He was lost to follow-up. However, 3 years later, he presented with recurrent visible haematuria. He had a computed tomography scan which revealed a mild-to-moderate dilation of the distal part of the right ureter and 0.8 cm thickening of the wall of the urinary bladder around the right ureteric orifice. There was no evidence of any significant lymph adenopathy or any other abnormal mass in the pelvis. He underwent transurethral biopsy of the thickened region and histological examination of the specimen revealed infiltration of lamina propria by ill-defined nested neoplastic cells with bland cytologic characteristics (see Figure 9). There was also evidence of perineural invasion by the tumour. The immunohistochemical staining characteristics of the tumour include immunoreactivity for high-molecular cytokeratin 903 and cytokeratin 7 and negativity for prostate specific antigen, prostate acid phosphatase, S100, and chromogranin. He subsequently underwent radical cystoprostatectomy elsewhere. The histology report from the institution where he underwent radical cystoprostatectomy stated that the tumour had extended through the lateral wall of the right trigone with vascular invasion and a focus of urothelial carcinoma *in situ*. The histology report also stated that in addition to the urinary bladder tumour there was focal adenocarcinoma of the prostate gland (Gleason pattern 3 + 3 = 6) which was confined to the left lobe of the prostate gland. The perivesical lymph nodes were negative for metastatic disease. This patient was lost to follow-up two months pursuant to his surgery.

Xiao and associates [28] stated that additional immunohistochemical stainings were done on the tumour specimens of the two patients and these showed that the nested neoplastic cells in both cases were strongly immunoreactive for p63 (a homolog of p53 protein) (Figures 8 and 10); 40% to 50% and 30% to 40% of tumour cells in Case  1 exhibited strong positivity for p53 and Ki-67, respectively, and no staining difference for either p53 or Ki-67 was present between superficial and deep infiltrating tumour cells. Focally positive stains for both p53 and Ki-67 were exhibited in the biopsy specimen of Case  2. The experience learnt from the biological behaviour of both Cases  1 and 2, especially in Case  1, would be indicative of the aggressive nature of nested variant of urothelial carcinoma.

Krishnamoorthy and associates [18] reported the case of a 46-year-old woman who presented with a two-year history of interrupted stream of urine on voiding. She developed acute urinary retention for which she was catheterized. Further evaluation after her catheterization revealed a large urinary bladder tumour. She did not have any history of haematuria or urinary tract infection. Her urine microscopy revealed 15 red blood cells per high power field. She had ultrasound scan of the abdomen which revealed bilateral mild hydroureteronephrosis up to the vesicoureteric junction. A huge heteroechoic pedunculated mass which measured 10 cm in size, with smooth surface and well-defined margin in the bladder, occupying the entire surface of the urinary bladder. She also had a contrast computed tomography scan of the abdomen which revealed normal liver, pancreas, and adrenals. The computed tomography scan also showed bilateral mild hydroureteronephrosis with a 10 mm simple cyst in the interpolar region of the right kidney. There was a large heterodense mass in the urinary bladder which occupied most of the bladder. There were multiple enlarged lymph nodes involving the parailiac right external iliac, left internal iliac, and left inguinal regions, each measuring from 7 mm to 8 mm in size in transverse diameter. She underwent cystoscopy which revealed a solid 10 cm × 10 cm pedunculated lesion arising from the right lateral and anterior wall of the bladder, which was a rounded, mobile, well-circumscribed tumour with a smooth surface. The mucosa over the mass lesion and the adjoining bladder surface appeared intact. The right ureteric orifice was not seen and the left ureteric orifice looked normal. The tumour was bimanually palpable and mobile. She underwent transurethral resection of the tumour and histological examination of the specimen was reported to have shown nested variant of transitional cell carcinoma with marked atypical epithelial proliferation. Microscopic examination of the tumour revealed that the entire tumour was infiltrated by nests of polygonal cells with oval vesicular to hyperchromatic nuclei and eosinophilic to clear cytoplasm. Cystically dilated cells were also seen. The intervening stroma consisted of spindle cells with fusiform nuclei, compressing the cell nests in some areas, forming broad polypoid projections. There were areas of necrosis with acute inflammatory reaction. The metaplastic stromal cells exhibited no increase in mitotic activity. Krishnamoorthy and associates reported this case at a stage when there was no follow-up information regarding the outcome of the patient. Krishnamoorthy and associates [18] stated the following.

- Nested variant of urothelial carcinoma can easily be confused with a number of benign lesions; it is very important for the pathologist to consider nested variant of urothelial carcinoma in the differential diagnosis of the lesions that show nested type growth pattern in lesions of the urinary bladder. It is equally important for the treating physician to adopt an aggressive approach towards the management of these lesions.
- The optimal treatment for nested variant of urothelial carcinoma is yet to be determined and this may be because of the rarity of the tumour, very small number of long-term survivors, and the absence of any randomized studies. The aggressive invasive growth and early metastases are the factors that favour radical cystectomy with adjunctive systemic chemotherapy. Nevertheless, a consensus is yet to be arrived at.
- They had reported their case in view of its rarity, its unusual histology, and its prognostic significance emphasizing the need to distinguish it from the classic transitional cell carcinoma.
- The aggressive behaviour of these nested variants underlines the importance of distinguishing them from benign proliferative lesions. Cytologic atypia is not a very good parameter because the mild atypia seen in nested variant of transitional cell carcinoma can be very deceptive, especially at low and medium power magnifications. Though the obvious invasion of the muscularis propria excludes the possibility of a benign lesion, the absence of invasion leads the pathologist onto a diagnostic dilemma.

Ooi and associates [21] reported a rare presentation of nested variant of transitional cell carcinoma in a 74-year-old man who had bilateral hydronephrosis and acute renal failure. At cystoscopy, both ureters were obstructed with the right ureter narrowed along the entire length. Subsequent histopathologic examination from the ureteral resection revealed nested variant of urothelial carcinoma. Bilateral stents were inserted and the patient survived 12 months with a good partial response to chemotherapy. They stated that at the time of the report of their case in 2006, 76 cases of nested variant of urothelial carcinoma had been reported in the literature and at that time their patient was the first, to their knowledge, to present with bilateral hydronephrosis and tumour extension along one ureter. He had extensive liver and bony metastases and he eventually died 12 months pursuant to the establishment of the diagnosis. Ooi and associates [21] stated that even though anecdotal reports of adjunctive chemotherapy with gemcitabine and carboplatin are being done, there are no available randomized studies on the effects of adjunctive chemotherapy in these patients with nested variant of urothelial carcinoma, after cystectomy. Furthermore, Holmäng and Johansson [20] reported that there was no survival advantage with adjunctive radiotherapy in their series of seven patients with T stage in cystectomy specimens ranging from T1 to T4B.

Holmäng and Johansson [20] stipulated that nested variant of transitional cell carcinoma is aggressive and invasive, with a very well-differentiated histology, which is difficult to understand. Nevertheless, it had been postulated that the unusual histology may be due to the peculiarities of the host response mechanisms to carcinogenic stimulus such that the host is able to channel differentiation but cannot control invasion.

Tatsura and associates [12] stated that the nested variant of transitional cell carcinoma has the characteristics of a focus of nests of transitional epithelial cells which infiltrate the lamina propria with apparent involvement of bladder mucosa. They also suggested that immunohistochemical analysis may help in the diagnosis of nested variants of transitional cell carcinoma derived from epithelial cells and that diagnosis and treatment at an early stage should reduce the mortality of patients with nested variant of transitional cell carcinoma.

Murphy and Deana [4] stated that the tumour cells of nested variant of transitional cell carcinoma are organized in nested structures and that many tumour cells are only slightly atypical, but a careful examination revealed that at least some significantly anaplastic cells are identifiable in each case, and the degree of anaplasia has a tendency to parallel the depth of invasion. They additionally stated that the features that identify this lesion as malignant are the tendency for increasing cellular anaplasia in the deeper portions of the lesion, its infiltrative nature, and the presence of muscle invasion. Mai and associates [41] stated that despite the presence of mild or minimal cytological atypia in nested variant of transitional cell carcinoma, these neoplasms are occasionally associated with an aggressive clinical course and even death.

Drew and associates [10] reviewed the clinicopathologic features of 16 nested variants of transitional cell carcinoma over a 13-year period. They reported the following.

- Nested variant of transitional cell carcinoma was characterized by the presence of irregular nests and/or tubules of transitional cells infiltrating the lamina propria without surface involvement.
- The neoplastic cells tended to have innocuous features but at least a few cells in every case are cytologically anaplastic.
- There was a marked male predominance.
- Synchronous or metachronous transitional cell carcinomas of more usual histologic make-up may occur.
- After a follow-up averaging 16.6 months, only three patients were known to be alive with no evidence of disease.

Drew and associates [10] in 1996 made the ensuing concluding iteration.

Clinicopathologic information from their 16 cases combined with the 8 cases of nested variant of transitional cell carcinoma that were reported before the publication of their paper confirms that nested variant of transitional cell carcinoma is a persistent and aggressive neoplasm that is notable for its innocuous appearance in histologic preparations.

Liedberg and associates [31] reported three cases of the nested variant of urothelial carcinoma that were treated in their institution. They compared their outcome data with those of previously reported cases. They reported that the three patients presented with advanced muscle-invasive nested variant of urothelial carcinoma, of which two had lymph node metastasis at cystoprostatectomy. The histopathology in the latter two cases showed the same picture in the lymph node as in the primary tumour with nests of tumour cells with mild-to-moderate atypia. In all three cases the tumour involved the ureteric orifice or bladder neck. They concluded the following.

- Nested variant of urothelial carcinoma is a rare but an important histopathologic entity.
- Nested variant of urothelial carcinoma has a poor prognosis.
- At an early stage, the tumours might be difficult to differentiate from benign conditions and awareness of this condition is of outermost importance.

Because of the rarity of nested variant of urothelial carcinoma most urologists and pathologists would not have encountered a case of nested variant of urothelial carcinoma before and as a result of this there is the possibility that a case of nested variant of urothelial carcinoma may inadvertently be misdiagnosed. It is therefore pertinent to document iterations of Dhall et al. [2], which summarize the microscopic features of nested variant of urothelial carcinoma as follows.

**Figure 4:**
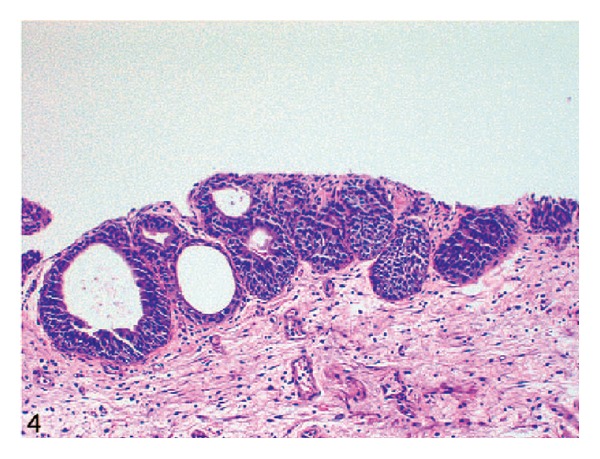
von Brunn nests in comparison with nested variant of urothelial carcinoma showing regularly spaced urothelial nests with a relatively flat base (hematoxylin-eosin, original magnification ×200). Nested variant of urothelial carcinoma taken from Dhall et al. [2]. The figures have been reproduced with the permission of the editor in chief of Archives of Pathology and Laboratory Medicine on behalf of the editorial board of the journal.

**Figure 5:**
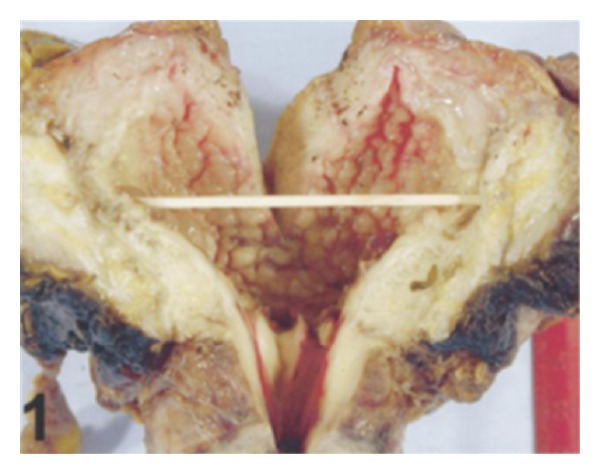
Gross photo of Case  1 shows an edematous urinary bladder mucosa and markedly and diffusely thickened bladder wall Xiao et al. [28].

**Figure 6:**
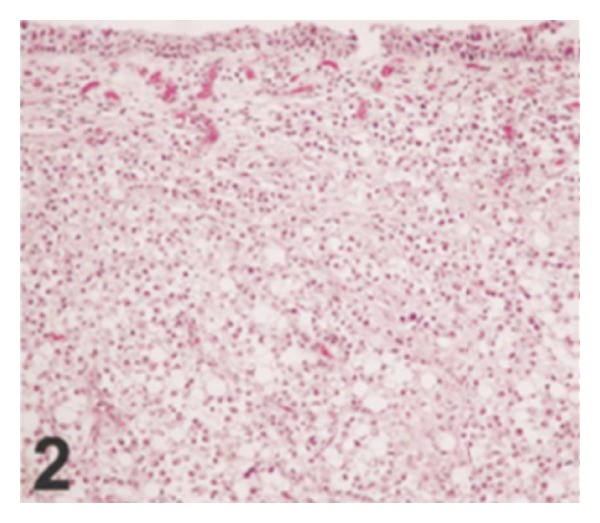
The neoplastic cells form ill-defined nests with a diffuse growth pattern. Some tumor cells have clear cytoplasm. The surface mucosa is not involved by the underlying tumor (Case  1) (hematoxylin-eosin, original magnification ×200) Xiao et al. [28].

**Figure 7:**
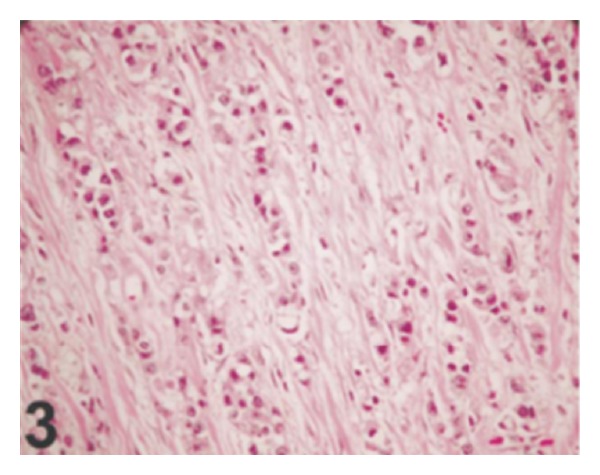
The nuclei of the tumor cells are relatively uniform with finely granular chromatin, inconspicuous nucleoli, and rare mitosis (Case  1) (hematoxylin-eosin, original magnification ×400) Xiao et al. [28].

**Figure 8:**
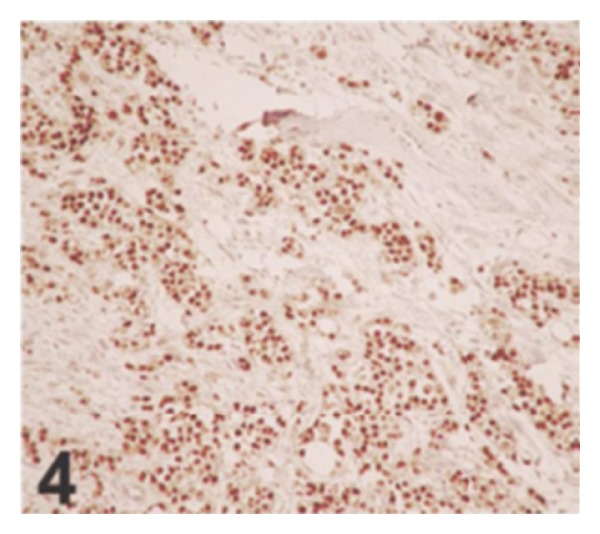
The neoplastic cells are strongly immunoreactive for p63 (Case  1) (p63, original magnification ×200) Xiao et al. [28].

**Figure 9:**
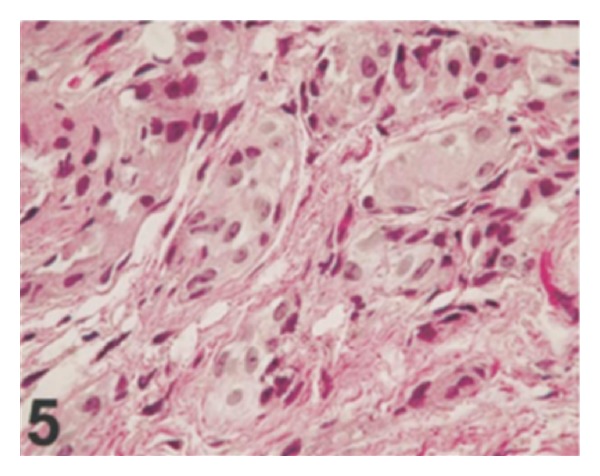
The cytologically bland neoplastic cells are arranged in a diffuse pattern of relatively ill-defined and variably sized nests (Case  2) (hematoxylin-eosin, original magnification ×600) Xiao et al. [28].

**Figure 10:**
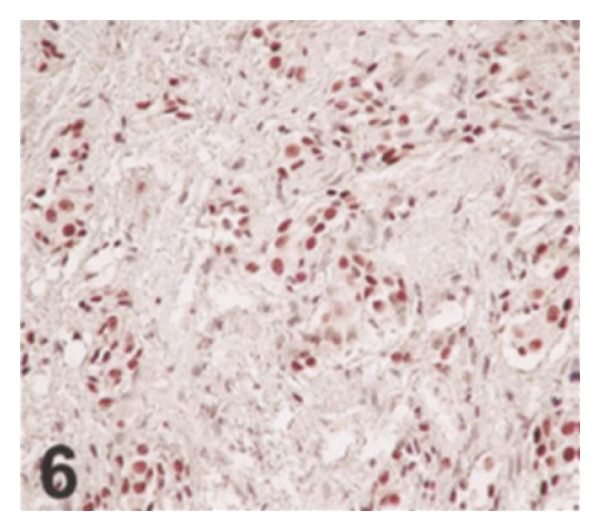
The neoplastic cells are strongly immunoreactive for p63 (Case  2) (p63, original magnification ×400) Xiao et al. [28].

- These tumours are characterized histologically by large numbers of small, closely packed, poorly defined, confluent, and irregular nests of uniform urothelial cells infiltrating the lamina propria, reminiscent of von Brunn nests, and also infiltrating the muscularis propria with retained nested pattern (see Figures 1 and 2).
- These nests exhibit an infiltrative base as described by Volmar et al. [30].
- Small tubules and microcysts may be seen as described by Talbert and Young [3] and Young and Oliva [9].
- The overlying urothelium may be normal in appearance.
- The cells comprising nested variant of urothelial carcinoma exhibit no significant cytologic atypia; they are mildly pleomorphic and show slightly increased nuclear-cytoplasmic ratio and occasionally prominent nucleoli (see Figure 3).
- Even though nested variant of urothelial carcinoma cells appears to be histologically bland, a number of authors [4, 10, 20] have observed significant pleomorphism, particularly within regions of muscle invasion.
- Mitotic figures are not readily seen. Mucin is not identified. The surrounding stroma varies from dense and collagenous to loose and myxoid or even oedematous. Lymphatic invasion may be seen [20].
- In view of their deceptively bland appearance, the tumours are sometimes misdiagnosed as benign lesions, especially in the biopsy material leading in some instances to a significant delay in the establishment of diagnosis as stated by Young and Olive [9]. In some instances it is very difficult to establish an unequivocal diagnosis of nested variant of urothelial carcinoma in the biopsy material until multiple biopsies are performed.
- Nested variant of urothelial carcinoma must be differentiated from the benign proliferative lesions of the urothelium, such as von Brunn nests, cystitis cystica, cystitis glandularis, nephrogenic adenoma, paraganglioma, and inverted papilloma (see Figure 4 which illustrates von Brunn nests in comparison with nested variant of urothelial carcinoma showing regularly spaced urothelial nests with a relatively flat base).

Dhall and associates [2] stated that the optimal treatment of nested variant of urothelial carcinoma is yet to be determined in view of the rarity of the tumour and in view of absence of randomized studies. They suggested that nested variant of urothelial carcinoma should be approached clinically as a high-grade disease with early cystectomy as an option for pT1 and pT2 tumours [2]. Dhall and associates [2] additionally stated the following that.

- Adjuvant chemotherapy and radiation therapy have not been shown by a number of authors to be significantly beneficial in their reported series [12, 20, 28].
- Nested variant of urothelial carcinoma should be kept in mind as a histologically unique variant which should not be confused with von Brunn's nest. Any bladder biopsy with tightly packed nests with any degree of architectural or cytological atypia should be evaluated with caution, and the possibility of nested variant of urothelial carcinoma should be raised in such circumstances.

Linder and associates [22] evaluated the oncological outcomes after radical cystectomy in patients with nested variant of urothelial carcinoma and compared survival to that in patients with pure urothelial carcinoma of the bladder. Linder and associates [22] identified 52 patients with nested variant of urothelial carcinoma of the urinary bladder who were treated with radical cystectomy between 1980 and 2004. The pathological specimens were rereviewed by a single genitourinary pathologist. The patients were matched 1 : 2 by age, gender, ECOG (Eastern COOperative Oncology Group) performance status, pathological tumour stage, and nodal status to patients with pure urothelial carcinoma. Survival was estimated using the Kaplan-Meier method and compared with the log rank test. Linder and associates [22] reported that the patients with nested variant of urothelial carcinoma of the urinary bladder had a median age of 69.5 years (IQR 62, 74) and a median postoperative follow-up of 10.8 years (IQR 9.3, 11.2). They also reported that nested variant cancer was associated with a high rate of adverse pathological features since 36 patients (69%) had pT3-pT4 disease and 10 (19%) had nodal invasion. Eight patients (15%) with nested variant cancer received preoperative chemotherapy. When the patients with the nested variant were matched to a cohort with pure urothelial carcinoma, no significant differences were noted in 10-year local recurrence-free survival (83% versus 80%, *P* = 0.46) or 10-year cancer specific survival (41% versus 46%, *P* = 0.75). Linder and associates [22] concluded that the nested variant of urothelial carcinoma is associated with a high rate of locally advanced disease at radical cystectomy. However, when stage matched to patients with pure urothelial carcinoma, patients with the nested variant did not have an increased rate of recurrence or adverse survival. Linder and associates [22] iterated that further studies are required to validate these findings and guide the optimal multimodal treatment approach to these patients.

Tripodi et al. [23] reported the case of a 49-year-old woman affected by hepatitis C virus who presented with fever, discomfort, urgency, and hypertension. She had a computed tomography scan which showed a sclerosing inflammatory process that involved the connective and adipose tissue of the renal sinus. In absence of renal or pelvic masses felt that an underlying malignancy was excluded and renal abscess or tuberculosis was suspected. Accordingly, nephrectomy and proximal ureterectomy was performed. Tripodi et al. [23] reported the following.

- Grossly, the calices, renal pelvis, and pelviureteric junction appeared modestly dilated with whitish, thickened, and uneven mucosa.
- Microscopic examination revealed that the subepithelial connective tissue, the fibromuscular layer, and the renal sinus fat were diffusely infiltrated by small nests of medium to large urothelial cells which were immunohistochemically stained positively with p63 and they had abundant eosinophilic cytoplasm and slightly atypical nuclei.

Tripodi et al. [23] stated the following.

- On the basis of morphologic and immunohistochemical features, a diagnosis of nested variant of urothelial carcinoma was made.
- After surgery, the patient recovered from hypertension.
- Pelvic and upper urothelial tract nested variant of urothelial carcinoma was uncommon, and to the best of their knowledge, their case was the second case of nested variant of urothelial carcinoma with renal pelvis involvement.

Cerda et al. [24] submitted an abstract for a poster presentation at the 25th European Congress of Pathology in Lisbon, Portugal (August 31st, to September 4th, 2013). They reported the case of 2 patients with nested variant of urothelial carcinoma as follows.

*Case 1.* A 53-year-old man presented with visible haematuria. He had ultrasound scan which showed an exophytic lesion in the urinary bladder. The patient underwent transurethral resection of bladder tumour which was reported on histological examination to be invasive urothelial carcinoma (pT2). He underwent, 2 months later, radical cystoprostatectomy (and the tumour was staged pT3b pN1), radiotherapy, and chemotherapy. The patient died one year later as a result of advanced metastatic disease.

*Case 2.* An 83-year-old woman who presented with asymptomatic visible haematuria underwent transurethral resection of bladder tumour, which on histological examination was reported to be an invasive urothelial carcinoma (pT2). Because of her advanced stage she did not undergo any surgical procedure. She died 3 years later.

With regard to the results, both tumours were reported to have shown similar histological features; the neoplastic cells were grouped in confluent smalls nests and abortive tubules which were composed of urothelial cells with nuclear atypia infiltrating deeply the wall of the urinary bladder and in Case  1, the perivesical tissue, urethra, and one lymph node were invaded. Immunohistochemistry profile revealed positive staining for CK7, CK20, 34*β*E12, and p63.

Yildiz et al. [25] reported the case of a 60-year-old man in a Turkish Journal, who presented with visible haematuria. Histologically, the tumour was characterized by irregular nests and small tubules of urothelial carcinoma cells which had infiltrated the lamina propria and deeper layers without involvement of the mucosal layer. Many of the tumour cells were only slightly atypical, but careful examination revealed at least some significantly anaplastic cells; the degree of cellular atypia tended to parallel the depth of invasion. They stated that the tumour tended to be aggressive despite the initial impression of a benign vascular lesion resembling a capillary haemangioma.

Pusztaszeri et al. [26] reported a case of nested variant of urothelial carcinoma of the renal pelvis and ureter which was synchronous with high-grade urothelial papillary carcinoma.

Lau [27] reported the case of a 71-year-old woman who had nested variant of urothelial carcinoma of renal pelvis. Lau [27] stated that the tumour was characterized by a nested pattern of growth and relatively bland cytologic features. The patient presented with a locally advanced disease at the time of nephroureterectomy.

## 4. Summary

Nested variant of urothelial carcinoma is a rare tumour and to the author's knowledge about 200 cases have so far been reported.

The tumour usually manifests at an advanced stage and tends to exhibit a persistent and progressive clinical course.

Death rate from nested variant of urothelial carcinoma can be up to 25% of cases and persistent or progressive disease has been reported in up to 60% of cases which had led some authors [10] to conclude that nested variant of urothelial carcinoma has a clinical course which is similar to that of high-grade urothelial carcinomas.

It is important to keep in mind nested variant of urothelial carcinoma as a unique histologic variant which should not be mistaken for florid von Brunn nest.

In cases where bladder biopsy exhibits tightly packed nests with any degree of cytologic atypia or architectural atypia, the biopsy specimens need to be evaluated with care in order to ascertain and exclude the possibility of nested variant of urothelial carcinoma.

Even though reports from case reports had indicated that the prognosis of nested variant of urothelial carcinoma is poor following treatment results of a recent study revealed that there is no significant difference between patients with nested variant of urothelial carcinoma and those with comparatively staged conventional urothelial carcinoma who had undergone cystectomy.

## 5. Conclusions

Nested variant of urothelial carcinoma is a rare tumour with characteristic histopathologic features which must be carefully identified to establish its diagnosis.

Previous case reports and case series indicated that nested variant of urothelial carcinoma tends to be diagnosed at an advanced stage and is associated with poor prognosis; however, results of a recent study revealed no statistically significant difference between the outcome of patients with nested variant of urothelial carcinoma and patients with conventional urothelial carcinoma of similar stages who had undergone cystectomy.

Correct and early diagnosis of this tumour is essential in order to provide early curative treatment in order to avoid diagnosis at an advanced stage.

There is the need for a multicentre trial to validate this recent finding and to identify treatment protocols that would help improve the outcome of the tumour following treatment.

## Figures and Tables

**Table 1:** A list of some of the reported cases of nested variant of urothelial carcinoma and their outcome.

Patient	Reference	Age/sex	Pathologic stage	AJCC stage	Treatment	Status	Follow-up	Associated CUC (WHO/ISUP 1998)
1	Lin et al. [7]	90/F	pT1	1	Cystectomy	NED	30	No

2	Lin et al. [7]	73/F	pT3b	III	Cystectomy + chemotherapy	NED	5	No

3	Lin et al. [7]	65/M	pT2b	IV	Cystectomy + chemotherapy	AWM	16	Flat CIS

4	Lin et al. [7]	70/M	pT3b	III	Cystectomy + chemotherapy	AWM	15	Flat CIS

5	Lin et al. [7]	61/M	pT3b	IV	Cystectomy + chemotherapy	NED	19	Flat CIS

6	Lin et al. [7]	80/M	pT3b	III	Cystectomy	NED	27	Flat CIS

7	Lin et al. [7]	66/M	pT1	I	Cystectomy	NED	22	No

8	Lin et al. [7]	53/M	pT1	I	Cystectomy	NED	19	No

9	Lin et al. [7]	51/M	pT2	IV	Radiotherapy	DOD	3	No

10	Lin et al. [7]	59/M	pT2a	II	Cystectomy	NED	24	No

11	Lin et al. [7]	42/F	pT2a	II	Cystectomy	NED	20	No

12	Lin et al. [7]	75/M	pT2a	I	TUR	NED	12	Low-grade papillary UC

13	Wang et al. [11]	49/F	G3pT4M1		Partial cystectomy; en bloc resection of distal ileum, caecum; resection of transplanted pancreas; right salpingo-oophorectomy; repair of ileocolostomy anastomosis; chemotherapy	DOD	12 months	Plus conventional high-grade TCC

14	Tatsura et al. [12]	70/M	G3pT3b Nx M0	>III	Bladder adherent and cystectomy abandoned; open bladder total layer biopsy + bilateral ureterocutaneostomy; chemotherapy	Alive with disease	12 months	Small tumour atypical cells positive for cytokeratin

15	Terada [13]	80/F	pT1		TUR	Alive with no tumour	6 months	Atypical cells forming nests and tubules in lamina propria without surface urothelial involvement

16	Terada [13]	78/M	pT1		TUR	Alive with no tumour	15 months	Atypical cells forming nests and tubules in lamina propria without surface urothelial involvement

17	De Berardinis et al. [14]	70/M	pT2aN+		TUR twice + BCG for initial conventional G3pT1b tumour and cystectomy + lymphadenectomy + chemotherapy for nested variant was started	Alive 12 months after initial diagnosis of conventional urothelial carcinoma	12 months after initial diagnosis and exact duration of follow-up after cystectomy not stated but patient just started on chemotherapy after cystectomy	

18, 19, 20, 21, 22, 23, 24	Cardillo et al. [15]	5 M 2 F 7 cases Age range 53–90 years		3 stage 1; 2 stage II; 1 stage III; 1 stage IV with metastasis	6 patients with stages I to III tumours underwent cystectomy; 1 patient with stage IV tumour had radiotherapy	Follow-up outcome data not provided	Follow-up outcome data not provided in paper	

25	Dundar et al. [16]	78/M	pT4N1		Cystoprostatectomy and chemotherapy planned but not given at time of publication	Alive	Follow-up data not available at time of publication	

26	Dundar et al. [16]	56/M	pT2 at least		TURBT he refused radical cystoprostatectomy	Alive but had 3 recurrences of nested variant urothelial carcinoma resected over 23 months	23 months	

27	Badoual et al. [17]	Details not available	Details not available in English	Details not available	Details not available to author	Details not available	Details not available	Details not available

28	Badoual et al. [17]	Details not available	Details not available	Details not available	Details not available	Details not available	Details not available	Details not available

29	Krishnamoorthy et al. [18]	45 F	pT1		TUR	Alive	No follow-up paper soon after TUR	

30	Ozdemir et al. [19]	65 M	pT2 at least		TUR biopsy Further details of treatment not mentioned in paper	Alive no data provided on follow-up case reported after biopsy	Data not available	

31	Holmäng and Johansson 2001 [20]	10 patients	Details not available to author		Details not available to author	7 died of disease or treatment complications (4 months–40 months); 1 died of unrelated cause after 90 months; follow-up ≤1 year for the remaining 2		

32	Ooi et al. [21]	74/M		Bladder tumour with ureteric involvement	TUR + bilateral local resection of ureter and chemotherapy	Alive with tumour (partial response)	12 months	

33–86	Linder et al. [22]	52 patients Mean age 69.5 years Range 62–74	36 (69%) with pT3-T4 10 (19%) with nodal invasion		All had radical cystectomy 8 had additional perioperative (15%) chemotherapy	Analysis in the comparison of patients with nested variant matched with a cohort of conventional urothelial carcinoma showed no significant differences in the 10-year local recurrence free survival (83% versus 80%, *P* = 0.46) or 10-year cancer specific survival (41% versus 46%, *P* = 0.75)	Median follow-up 10.8 months Range 9.3–11.3	

87	Tripodi et al. [23]	49/F	Renal pelvis pT1		Nephrectomy and upper ureterectomy	Alive	No long-term follow-up at time of publication	

88	Cerda et al. [24]	53/M	pT3b pN1		TUR and radical cystoprostatectomy, radiotherapy, and chemotherapy	Died due to advanced metastatic disease	12 months	

89	Cerda et al. [24]	83/F	pT2		TUR (surgical procedure not done due to advanced age)	Died of disease	3 years (36 months)	

90	Yildiz et al. [25]	60/M	T2 at least (muscle invasion)		TUR further details in Turkish language	Not found in data		

91	Pusztaszeri et al. [26]		Renal pelvis and ureter		Details not available to author	Details not available to author	Details not available to author	

92	Lau [27]		Renal pelvis	Locally advanced	Details not available to author	Details not available to author	Details not available to author	

93	Stern [1]	45/M	Details not available to author	Details not available to author	TUR	Alive tumour recurred 18 months later	18 months	

94, 95, 96	Talbert and Young [3]	3 men Aged 53–73 years	Details not available to author		Details not available to author	Details not available to author		

97, 98, 99, 100	Murphy and Deana [4]	4 cases	Details not available to author	Details not available to author	Details not available to author	Tumours persisted/recurred	Details not available to author	

101–130	Wasco et al. [8]	30 cases Aged 41–83 years Average 63 Male-female 2.3 : 1	All but 1 invasive tumours (9% pT1; pT2-3a; 65% pT3b; 17% pT4)		All had cystectomy and 15 had cystectomy and chemotherapy	3 (10%) died of disease; 16 (55%) alive with persistent or recurrent disease; 10 (34%) alive without disease Response to chemotherapy observed in 2 (13%) of 15 patients	Follow-up in 29 patients (97%) with median 12 months (range 1–31 months)	Focal atypia in 90% and focal high-grade cytological atypia at tumour base in 40%

131–134	Young and Oliva [9]	4 patients Age range 70–85 years	Details not available to author		Details not available to author	Details not available to author		1 or more specimens were misinterpreted as benign

135–150	Drew et al. [10]	16 cases with marked male predominance	Details not available to author		Details not available to author	3 with no disease	Average follow-up 16.6 months	A few cells in every case with cytological atypia

151 and 152	Xiao et al. [28]	69/M 70/M	pT4 N1 pT2/3 + perineural and vascular invasion + Gleason 3 + 3 adenoca of prostate		Radical cystoprostatectomy and chemotherapy Radical cystoprostatectomy	Developed bone and soft tissue metastases in 4 months	4 months Lost to follow-up at 2 months	Focal urothelial CIS and multiple foci of urothelial atypia Focus of urothelial CIS

153	Huang et al. [29]	83/M	G3pT2-3		Cystoprostatectomy	Alive no long-term follow-up data available to author		

154–164	Volmar et al. [30]	11 patients	5 pT2-3 N0 1 pT2-3 M1; 1 pT4 stage of remaining not available to author			Details not available to author	Details not available	

165–174	Holmäng and Johansson [20]	10 patients	Details not available		Locoregional therapy	7 died of disease or treatment complications 4–40 months after diagnosis; 1 died of unrelated cause after 90 months; Follow-up ≤1 year for the remaining 2		

175–177	Liedberg et al. [31]	3 patients		Advanced muscle-invasive 2 with lymph node involvement	Final outcome not available			

178–200	Cox and Epstein [32]	23 cases Mean age 63.7 years; Range 39–89; 86% male	20 T2-T3 2 pT1 1a pT1b		18 had TURBT 2 nephroureterectomy 3 radical cystectomy	3 of 17 patients developed metastasis 2 lung 1 unknown with 2 of the 3 dead of disease; 1 patient died of disease with no known details	Follow-up for 17 patients Mean 43 months Range 5 months to 9 years	

Abbreviations: M: male; F: female; NED: alive with no evidence of disease; AWM: alive with metastasis; DOD: dead of disease; CIS: carcinoma *in situ*; CUC: conventional urothelial carcinoma; TUR: transurethral resection of tumour; and Lin et al.: Lin O, Cardillo M, Dalbagni G, Linkov I, Hutchinson B, and Reuter V E. Nested variant of urothelial carcinoma: a clinicopathologic and immunohistochemical study of 12 cases. Modern Pathology 2003; 16(12): 1289-1298.

**Table 2:** Nested variant of urothelial carcinoma: example of results and geographic distribution of several markers in nested variant of urothelial carcinoma taken from Lin O, Cardillo M, Dalbagni G, Linkov I, Hutchinson B, and Reuter V E. Nested variant of urothelial carcinoma: a clinicopathologic and immunohistochemical study of 12 cases. Modern Pathology 2003; 16(12): 1289–1298.

Case	Ref.	P21 result	P21 location	P27 result	P27 location	P53 result	P53 location	EGF-R result	Bc12 result	Bc12 location	MIB-1%	MIB1 location	Other comments
1	Lin et al. [7]	+	Diffuse	+	Surface	−		−	−		20	Diffuse	

2	Lin et al. [7]	+	Diffuse	+	Diffuse	−		−	−		15	Diffuse	

3	Lin et al. [7]	+	Diffuse	−		−		−	−		35	Diffuse	

4	Lin et al.[7]	+	Diffuse	+	Surface	−		−	−		35	Diffuse	

5	Lin et al. [7]	+	Base	+	Surface	+	Base	−	+	Surface	2	Base	

6	Lin et al. [7]	−		+	Surface	−		−	−		20	Diffuse	

7	Lin et al. [7]	+	Base	+	Surface	−		−	+	Surface	35	Diffuse	

8	Lin et al. [7]	+	Base	+	Surface	−		−	−		15	Base	

9	Lin et al. [7]	−		+	Surface	+	Diffuse	−	−		30	Diffuse	

10	Lin et al. [7]	+	Diffuse	+	Surface	−		−	−		30	Base	

11	Lin et al. [7]	+	Base	+	Surface	−		−	−		20	Diffuse	

12	Lin et al. [7]	+	Base	+	Surface	+	Base	−	−		15	Base	

13	Wang et al. [11]	Not done		Not done		−		Not done	Not done		Not done		

14	Tatsura et al. [12]	Not done		Not done		Not done		Not done	Not done		Not done		Cytokeratin+

15	Terada [13]	Not done				+		Not done					Ki-67 labeling = 15%; +cytokeratins; +EMA; *α*-methylacyl CoA racemase; +Ca19-9; MUC1

16	Terada [13]	Not done		Not done		+		Not done					Ki-67 labeling = 30%; +cytokeratins; +EMA, +p63, +p53, +C10, +CEA, +MUC1

17	De Berardinis et al. [14]	Not done		Not done		Strongly+		Not done					Ki-67 +high expression

18, 19, 20, 21, 22, 23, 24	Cardillo et al. [15] (7 cases)	Not done		Not done		Not done		Not done					

25	Dundar et al. [16]	Not done		+40%		+40%		Not done					Ki-67 20%; 34*β*E12; −PSA; −AE1

26	Dundar et al. [16]	Not done		+50%		+40%							Ki-67 15%; +34*β*E12; −PSA; −AE1

27	Badoual et al. [17]	Details not available	Details not available	Details not available	Details not available	Details not available	Details not available	Details not available	Details not available	Details not available	Details not available	Details not available	Details not available

28	Badoual et al. [17]	Details not available	Details not available	Details not available	Details not available	Details not available	Details not available	Details not available	Details not available	Details not available	Details not available	Details not available	Details not available

29	Krishnamoorthy et al. [18]	Details not available	Details not available	Details not available	Details not available	Details not available	Details not available	Details not available	Details not available	Details not available	Details not available	Details not available	Irregular nests of tubules in lamina propria

30.	Ozdemir et al. [19]	Not done		Not done		Not done		Not done				Not done	Details not available to author

31	Holmäng and Johansson [20]	Details not available to author	Deatials not available to author	Deatials not available to author	Deatials not available to author	Deatials not available to author	Deatials not available to author	Deatials not available to author	Deatials not available to author	Deatials not available to author	Deatials not available to author	Deatials not available to author	

32	Ooi et al. [21]	Details not available to author	Details not available to author	Details not available to author	Details not available to author	Details not available to author	Details not available to author	Details not available to author	Details not available to author	Details not available to author	Details not available to author	Details not available to author	

33 to 86	Linder et al. [22]	52 patients	Details not availabe	Details not availabe	Details not availabe	Details not availabe	Details not availabe	Details not availabe	Details not availabe	Details not availabe	Details not availabe	Details not availabe	

87	Tripodi et al. [23]	Not done		Not done		Not done							+P63

88	Cerda et al. [24]	Not done		Not done		Not done	Not done	Not done	Not done	Not done	Not done		+CK7,+CK20, +34*β*E12, +P63

89	Cerda et al. [24]	Not done	Not done	Not done	Not done	Not done	Not done	Not done	Not done	Not done	Not done		+CK7,+CK20, +34*β*E12, +P63

90	Yildiz et al. [25]	Details unavailable		Details unavailable		Details unavailable		Details unavailable		Details unavailable		Details unavailable	

91	Pusztaszeri et al. [26]	Details not available to author		Details not available to author		Details not available to author		Details not available to author		Details not available to author	Details not available to author	Details not available to author	

92	Lau [27]	Details not available to author	Details not available to author	Details not available to author	Details not available to author	Details not available to author	Details not available to author	Details not available to author	Details not available to author	Details not available to author	Details not available to author	Details not available to author	

93	Stern [1]	Details not available to author	Details not available to author	Details not available to author	Details not available to author	Details not available to author	Details not available to author	Details not available to author	Details not available to author	Details not available to author	Details not available to author	Details not available to author	

94–96	Talbert and Young [3]	Details not available to author	Details not available to author	Details not available to author	Details not available to author	Details not available to author	Details not available to author	Details not available to author	Details not available to author	Details not available to author	Details not available to author	Details not available to author	

97–100	Murphy and Deana [4]	Details not available to author	Details not available to author	Details not available to author	Details not available to author	Details not available to author	Details not available to author	Details not available to author	Details not available to author	Details not available to author	Details not available to author	Details not available to author	

101–130	Wasco et al. [8]												CK7+ (93%); CK20+ (68%); P63+ (92%); CK903+ (92%)

131–134	Young and Oliva [9]	Details not available to author	Details not available to author	Details not available to author	Details not available to author	Details not available to author	Details not available to author	Details not available to author	Details not available to author	Details not available to author	Details not available to author	Details not available to author	

135–150	Drew et al. [10]	Details not available to author	Details not available to author	Details not available to author	Details not available to author	Details not available to author	Details not available to author	Details not available to author	Details not available to author	Details not available to author	Details not available to author	Details not available to author	

151 and 152	Xiao et al. [28]					High +p53							Both Strongly+ for 63; and High Ki-67 indices Case 1 +CK 903; +CK7 +CK20; −PSA; −prostatic acid phosphatase; Strongly +p63; +p53 40% to 50%; +Ki-6730 to Case 2 +CK903; +CK7; −PSA; −prostatic acid phosphatase; −S100; −chromogranin; +p63 30 to 40%; Focally +p53; Focally +Ki-67

153	Huang et al. [29]												+CK7; +CK20; +thrombomodulin; +34*β*E12

154–164	Volmar et al. [30]										8.8%		+p53 4.2%; +P27 4.7%;

165–174	Holmäng and Johansson [20] 10 cases	Details not available	Details not available to author	Details not available to author	Details not available to author	Details not available to author	Details not available to author	Details not available to author	Details not available to author	Details not available to author	Details not available to author	Details not available to author	

175–177	Liedberg et al. [31]	Details not available	Details not available to author	Details not available to author	Details not available to author	Details not available to author	Details not available to author	Details not available to author	Details not available to author	Details not available to author	Details not available to author	Details not available to author	

178–200	Cox and Epstein [32]	Details not available	Details not available to author	Details not available to author	Details not available to author	Details not available to author	Details not available to author	Details not available to author	Details not available to author	Details not available to author	Details not available to author	Details not available to author	
